# Perceived Helpfulness of a Moderated Online Social Therapy Network for Young People Experiencing Social Anxiety

**DOI:** 10.3390/ijerph18062796

**Published:** 2021-03-10

**Authors:** Bridget O’Bree, Courtney C Walton, Sarah Bendall, Michael Wilson, Lee Valentine, Carla McEnery, Simon D’Alfonso, Mario Alvarez-Jimenez, Simon Rice

**Affiliations:** 1 School of Behavioural and Health Sciences, Australian Catholic University, Fitzroy, Victoria 3065, Australia; bridget.obree@myacu.edu.au; 2 Orygen, Parkville, Victoria 3052, Australia; courtney.walton@orygen.org.au (C.C.W.); sarah.bendall@orygen.org.au (S.B.); michael.wilson@orygen.org.au (M.W.); lee.valentine@orygen.org.au (L.V.); carla.mcenery@orygen.org.au (C.M.); dalfonso@unimelb.edu.au (S.D.); mario.alvarez@orygen.org.au (M.A.-J.); 3 Centre for Youth Mental Health, University of Melbourne, Parkville, Victoria 3052, Australia; 4 School of Computing and Information Systems, University of Melbourne, Parkville, Victoria 3052, Australia

**Keywords:** young people, social anxiety, digital interventions, qualitative evaluation, gender sensitisation

## Abstract

There is a growing need for more effective delivery of digital mental health interventions, particularly for individuals experiencing difficulty accessing or engaging with traditional face-to-face therapy. Young people with social anxiety, and young males with social anxiety in particular need interventions sensitized to their needs. While digital interventions for mental health have proliferated, increasing their accessibility and utility, the data on acceptability and effectiveness of these interventions, however, indicates a need for improvement. The current study sought to utilise qualitative data from semi-structured interviews with 70 participants (male *n* = 33; age range = 14–25 years, mean age = 19.8) from a single-group pilot study of a novel intervention for young people with social anxiety (Entourage), using a content analysis approach. Results indicated that participants spoke about five main categories: connection, anxiety management, appeal, disengagement and system improvement. No overt gender differences were found in the appeal or perceived helpfulness of the Entourage platform. The current study provides valuable information and suggestions to guide future improvement of digital interventions for young people, particularly those experiencing social anxiety.

## 1. Introduction

### 1.1. Social Anxiety Disorder and Young People

Social Anxiety Disorder (SAD) is one of the most common mental disorders affecting young people aged 12 to 25 years, with a mean onset during mid-late adolescence [1,2,3]. SAD is characterised by an intense fear of negative evaluation by others, particularly in social or performance situations, often resulting in thoughts and behaviours that maintain the disorder [3]. If left untreated, the disorder usually follows a chronic course, which can lead to significant consequences for those affected including comorbid depression, increased risk of suicidality, substance and alcohol dependence, academic underperformance, social isolation and poor social relationships [4,5,6]. This highlights the potentially severe impact SAD can have on a young person’s life trajectory and underscores the importance of early intervention for social anxiety symptoms.

Many young people with social anxiety never seek treatment for their difficulties, and if they do, it is often after many years of impaired functioning and comorbid psychiatric conditions [7,8,9]. Reasons for low help-seeking include symptoms of the disorder itself (e.g., fear of negative evaluation, perceived stigma), minimisation of symptom severity, and barriers such as financial and geographical restrictions limiting access to services [7]. From a gendered perspective, young men, in particular, tend to experience additional difficulty accessing in-person psychological support for social anxiety due to gender-specific stigma and conformity to social norms such as stoicism and self-reliance [10]. This highlights the important role effective and engaging digital health solutions could play for individuals with social anxiety more broadly, but young men in particular.

Digital interventions have been recognised as an increasingly powerful tool for intervention and prevention of mental health disorders as they offer increased accessibility, immediacy of care, self-directed engagement and anonymity which may be particularly appealing to those who face barriers to accessing traditional in-person support [11,12]. This has become especially important in the era of COVID-19 and restrictions placed on in-person supports, which could be an ongoing way of living. Additionally, young people spend much of their time using the internet for social networking and information seeking, therefore the delivery of mental health interventions via the internet may be particularly appealing to this population [13,14].

### 1.2. CBT-Based Digital Interventions

A recent systematic review and meta-analysis of digital interventions for depression and anxiety in young people reported a small effect in favour of digital interventions compared to waitlist controls (*g* = 0.45), showing CBT-based interventions to be most effective with a medium effect size (*g* = 0.66) [15]. Similarly, Podina et al. [16] found that digitally delivered CBT for children and adolescents was as effective as standard CBT (*g* = 0.295) and more effective than waitlist controls (*g* = 1.41) at reducing anxiety. For SAD, the therapeutic mechanisms in which CBT is purported to affect clinical outcomes is by specifically targeting both the symptoms and maintaining factors of the disorder [7,17,18]. Alongside preliminary evidence that digital interventions can reduce social anxiety symptoms, studies generally report participant satisfaction with existing interventions [15,19]. However, nearly all reported low treatment completion and high drop-out rates [15]. This suggests that while studies show promising early findings, treatment adherence and clinical outcomes are important areas for improvement. This is important as research suggests that greater treatment adherence or greater exposure to treatment material may lead to greater clinical improvements [20]. Previous studies have largely omitted collection of participants’ in-depth experiences of the engagement strategies used in digital interventions. This is essential information to garner given the necessary depth is often not captured by quantitative methods alone.

Grist, Croker, Denne and Stallard [15] found that the level of therapist support provided has a significant effect on trial effect sizes, reporting that therapist-guided digital interventions showed greater effect sizes than self-guided interventions. This suggests that the level of contact provided by a therapist can improve treatment adherence to digital interventions. Previous studies have also found that an element of human connection and support can enhance engagement with digital interventions [21]. In addition, there is growing evidence to suggest that peer support and social networking opportunities could improve the clinical effectiveness of digital interventions for SAD [14,22]. This is because social networking and peer support may help reduce feelings of loneliness among young people with SAD, as well as increase feelings of social connectedness which could serve as protective factors [23]. It is also important that social networks address barriers to engagement typically associated with individuals that experience SAD.

### 1.3. Program Design—Entourage

**Figure 1:**
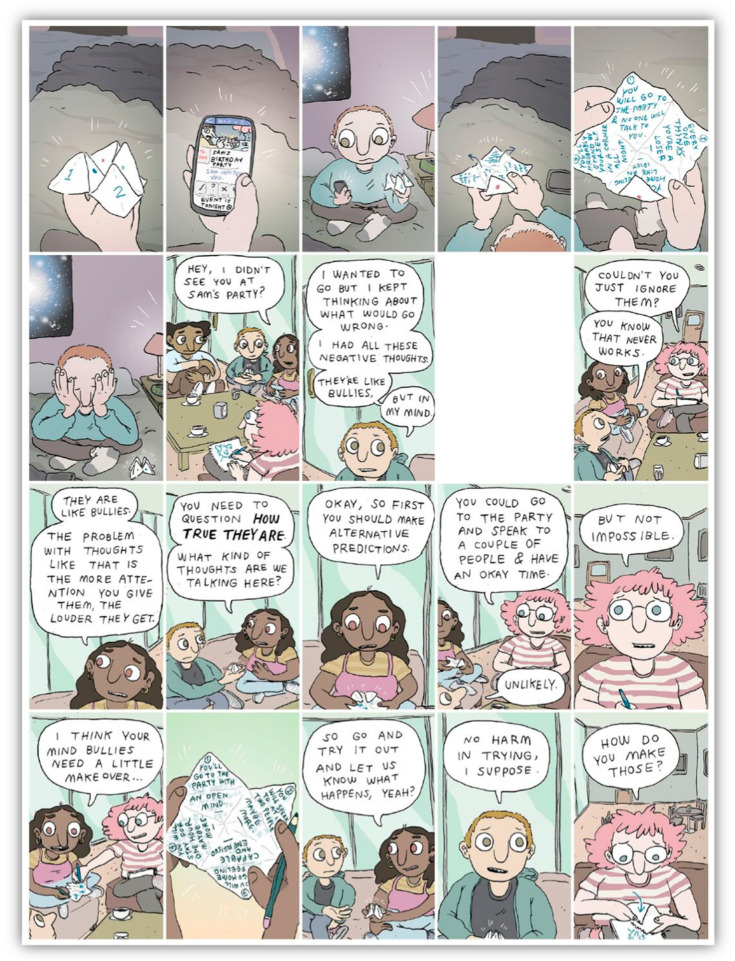
Example of a Graphic-Medicine Style Comic Used in the Entourage Platform.

To the authors’ knowledge, no trials exploring the efficacy of digital interventions for SAD have investigated the appeal or engagement of these platforms for different genders. This is important to consider as previous reports suggest males are less likely to be engaged in health services, including online interventions [12,24]. Therefore, understanding the experience of using a digital intervention by gender (and tailoring intervention accordingly) could provide important insights helping future interventions to increase engagement. Based on previous literature outlined above, a novel digital intervention for social anxiety was developed called Entourage with an emphasis on the engagement and treatment outcomes of young people, but especially young males. Entourage uses the Moderated Online Social Therapy (MOST) model of intervention that has been piloted in previous studies with young people, and their carers, experiencing a range of diagnoses [25,26,27,28,29]. The Entourage intervention is described in detail elsewhere, [30], however in brief, the MOST model uniquely integrates a social-networking aspect within the online platform in which participants are able to engage in peer-to-peer interactions and support [25,31]. This involves participants having access to evidence based psychosocial therapy content that is delivered via bespoke therapy comics (see Figure 1), as well as mindfulness and self-compassion audio tracks, opportunities for moderated group problem solving, interacting with peers in a safe online environment and access to support from trained mental health clinicians via chat functions. The Entourage platform design process included feedback from focus groups and consultations with a gender diverse group of young people who had or were currently experiencing social anxiety. The purpose of this was to a) include consumer perspectives in development and b) attempt to increase the engagement of young men. User experience and co-design with consumers of mental health interventions is recognised as an important component, with particular regard for program acceptability [32]. Clinical outcomes of the pilot study indicated significant and clinically meaningful improvements on a number of symptoms such as social anxiety, loneliness, social disconnectedness and wellbeing, with no gender differences found [30]. Similarly, usage data showed no differences in usage patterns between genders.

Rigorous evaluation of the Entourage platform is essential to understanding whether it has improved on the limitations identified in previous digital interventions for SAD. In order to do so, the use of qualitative methods to contextualise quantitative results are highly recommended [33,34,35]. For example, qualitative research methods can provide a richer understanding of the factors underpinning digital intervention use, and also provide insight into developing more acceptable and effective online programs for consumers of these services in the future [34,36].

### 1.4. Aims

The aim of the current study was to better understand the experiences of Entourage users and explore any gender differences in perceived helpfulness and other aspects of the platform using qualitative data generated from semi-structured interviews. The study also sought to understand the barriers and facilitators associated with ongoing engagement, and to gain insights into improving the acceptability and effectiveness of future digital interventions. These outcomes were considered especially important in the current climate (e.g., COVID-19), as technology progresses and the demand for digitally delivered support increases, which may be the only option available for some geographically and financially limited young people. An inductive reasoning approach was used to flexibly attend to the data in its context to generate conclusions about young peoples’ experiences. As the current study was designed as an exploratory investigation of participants’ subjective experiences, hypotheses were not developed in order to avoid biasing the inductive reasoning process.

## 2. Materials and Methods

### 2.1. Research Design

Participants of the 12-week Entourage project pilot study, described in detail elsewhere [30,37] were invited to share and reflect on their experiences of using the platform in semi-structured interviews. All participants interviewed had previously participated in the Entourage pilot study (Australian New Zealand Clinical Trials Registry: ACTRN12619000923167). Comprehensive analysis of outcomes for this trial are explored elsewhere [30]. Data reporting adhered to the Consolidated Criteria for Reporting Qualitative Research (CORE-Q) guidelines [38].

### 2.2. Participants

Semi-structured interviews were conducted with 70 participants, recruited from four headspace youth early-intervention centres in Melbourne, Australia. The participants who completed the follow-up interviews were a subset of the total participants who completed the Entourage pilot study (*n* = 86). Sixteen participants did not consent to participate in the feedback interview, reasons for declining were not recorded. Full study inclusion and exclusion criteria can be found in Appendix A.

### 2.3. Procedure

A full description of the study procedure can be found in the quantitative outcomes of the study [30]. Participants were referred by clinical staff at headspace to the study. They were then screened for eligibility, provided informed consent and completed a baseline assessment. After 12-weeks of receiving the intervention, participants were invited for a follow-up assessment, at which time the semi-structured feedback interviews were completed. The qualitative interviews were conducted between December 2018 and July 2019 by three research assistants, (BOB, MW and LV) who were supervised throughout the data collection process (SR). Details about interviewers including gender, credentials, experience and training can be viewed in Appendix B, however these details were not shared with participants and no relationships had been established prior to data collection. Only one research team member and a participant were present during the interviews. Participants could choose to have their interview audio-recorded or recorded manually and were reimbursed AUD $30 for completing each of the baseline and follow-up assessments. No interviews were repeated, and responses were recorded verbatim by the researcher during interviews if a participant did not consent to being recorded. Transcripts were not returned to participants to comment on or correct. Ethics approval was obtained from the University of Melbourne Human Research Ethics Committee (1851797).

### 2.4. Intervention

**Figure 2:**
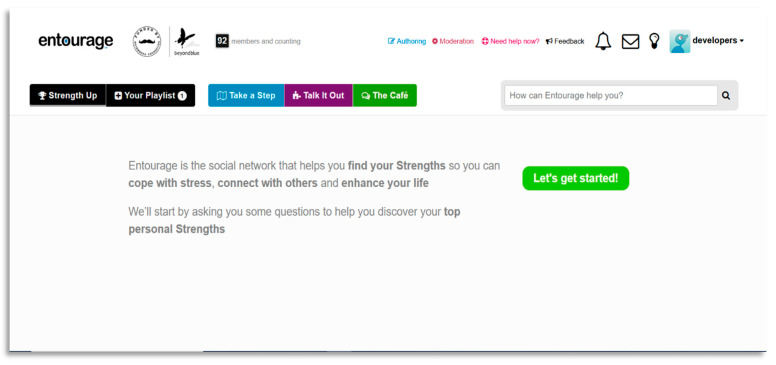
Welcome page of the Entourage platform.

**Figure 3:**
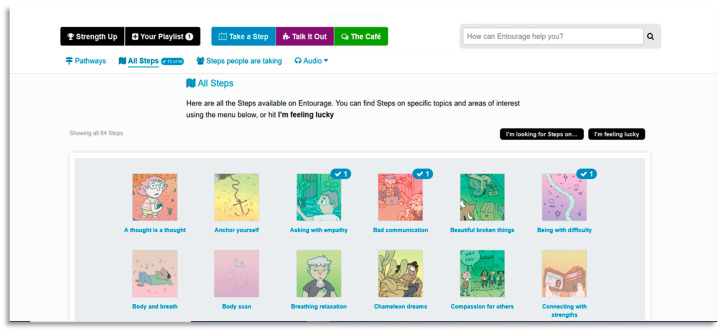
Participants’ view of available therapy “Steps” within the Entourage platform.

The Entourage intervention provided to participants during the trial represents an adaptation of the MOST model [25]. This included peer-to-peer social networking features where participants can interact with each other, psychosocial interventions delivered via interactive graphic medicine style comics, peer-support and expert clinical moderators who provide individualized support tailored to each user and ensure the safety of those using the platform [39]. The therapy content targeted cognitive and behavioural components of social anxiety via 12 individual modules called “steps” designed to be completed over a 12-week period. Alongside this was additional psychoeducational information on related issues such as depression, generalized anxiety, self-compassion, mindfulness and communication. An example of a bespoke therapy comic can be viewed in Figure 1, along with examples of the Entourage user interface in Figure 2 and Figure 3. The intervention was available to access as a web-based app and was only available to young people with symptoms of social anxiety who were clients of relevant headspace centres.

### 2.5. Data Collection

#### 2.5.1. Demographics/Quantitative Data

Following the provision of informed consent (including parental consent for those aged <18 years), participants were asked to provide contact details and baseline demographic information, alongside completion of quantitative baseline measures. The same measures were completed again at the 12-week follow-up assessment.

#### 2.5.2. Semi-Structured Interviews

The semi-structured interviews with Entourage participants sought to gain an understanding of young peoples’ experiences of using Entourage with particular reference to most and least beneficial aspects in addition to engagement and ideas for future improvements to the intervention. The interview schedule used by research assistants to guide interviews with Entourage participants and the research questions that guided question development are outlined in Appendix C. The interviews ranged from 3 to 25 min (*M* = 9 m 26 s, *SD* = 4 m 44 s). Interviewers were able to ask additional probing questions at their discretion due to the semi-structured nature of the interview schedule. Participants were typically interviewed by the same research assistant who completed all prior assessments with the young person, however this was not always the case and was dependent on researcher availability.

### 2.6. Qualitative Data Analysis

A structured and systematic content analysis was undertaken using the process outlined by Erlingsson and Brysiewicz [40] and detailed in Table 1. This method was used because it is a theoretically flexible, systematic and objective way of making replicable and valid inferences from a large amount of data to provide new insights and guide practical implications [41]. First, interview audio recordings were transcribed verbatim by an external provider. Transcripts were then read through to ensure familiarity, and notes were kept on any initial reflections on the data. Four transcripts were coded line-by-line in the margins of a Microsoft Word document by two researchers (BOB and CW) for meaning units, these meaning units were then assigned a code that captured the essential meaning of the phrase. This phase involved discussion until 100% agreement was reached on the codes, which subsequently informed the development of the coding frame. Following development of the coding frame, ten further transcripts were coded by BOB and CW and 100% agreement of coding reached, this totalled 20% of the data. The remaining transcripts were coded independently by author BOB and codes were checked by the research team to ensure accurate reflection of the overall dataset. Categories were then developed and refined via researcher triangulation and code frequencies were identified per case.

## 3. Results

A total of 70 participants aged 14–25 years (*M* = 19.8 years) participated in the study; 32 of the participants were male, 33 were female, and 3 were gender non-binary. Most participants were students (37.1%; *n* = 26) or working part time or casual (24.3%; *n* = 17) and 62.9% lived with parents or family (*n* = 44). In terms of education level, most had partially (35.7%, *n* = 25) or fully completed high school (28.6%, *n* = 20), with the remainder having completed some form of higher education. Twenty-six participants (37.1%) reported concurrent psychiatric medication at baseline, 26 (37.1%) reported no medication and 18 (25.7%) did not disclose this information. At post-treatment participants had completed an average of 3.5 concomitant in-person therapy sessions with an allied health practitioner at headspace centres. In terms of symptom severity, at baseline 10.0% of the sample reported mild social anxiety symptoms, 10.0% moderate symptoms, 15.7% marked symptoms, 15.7% severe and 38.6% very severe social anxiety symptoms as assessed by the Leibowitz Social Anxiety Scale (LSAS), a validated, gold-standard measure of SAD [42]. Comorbid depression symptoms were common across the sample with 8.6% with minimal depression symptoms; 15.7% mild depression; 32.9% moderate depression; 28.6% moderately severe depression; and 14.3% severe depression as measured by the Patient Health Questionnaire—9 a validated assessment of depression symptoms (PHQ-9) [43].

The sample was split into two groups to explore experiences of the Entourage platform: males (including trans-males) and non-males (i.e., female and gender non-binary young people). Gender was operationalized according to participant preferred gender identity, as opposed to sex assigned at birth. Five categories were derived from the data; these were: connection, anxiety management, appeal, disengagement, and system improvement. A full description of codes associated with overarching categories can be found in Table 2.

### 3.1. Connection

Connection referred to the experience of feeling connected to other individuals on the Entourage platform through shared experiences and understanding. It also referred to feeling supported and connected to the therapists and peer support workers who provided support on Entourage. Evident across participants of all genders was the finding that reading others’ descriptions of similar experiences was among the most beneficial aspects of Entourage (*n* = 33):

*I think, yeah, it came down to the fact that I wasn’t really alone in this, you know? Like, you could actually see and read the situations other people found themselves in and the ones that were similar to me and things like that so, I just like a sense of connection to the community of Entourage and I think that’s what I liked.*

(E230, Male, 23 years)

*Just like knowing other people are going through it as well. Because I really felt like it was like a one-person thing, like my own thing. But now like, I know some other people now, there’s like 80 that are feeling the same thing. So just having that in the back of my head just feels a bit better.*

(E008, Female, 14 years)

Participants reported that reading others’ experience of anxiety or mental ill-health helped to normalise their own experiences, reassuring them that they were not alone in their experience. This helped create a supportive online community.

### 3.2. Anxiety Management

Anxiety management referred to information, strategies and aspects of the site that were directly related to addressing or improving social anxiety symptoms. The vast majority of participants (*n* = 57) indicated that learning about social anxiety and anxiety management techniques was the most beneficial aspect of Entourage. Specifically, young people highlighted that having access to psychoeducational information about social anxiety, relatable examples of common situations and symptoms, and practical strategies for managing their anxiety was helpful. Young people spoke directly about their anxiety symptoms reducing and others that they had felt an increase in confidence when socialising after using the therapy material on Entourage.

*Gaining a lot more skills and knowledge about how I can change my thinking about certain things or do things and get through situations and how to communicate better and things like that gave me a lot of resources that I can still go back and use. *

(E229, Male, 17 years)

*I think just overall, I felt sort of when I went back out with into other social situations I felt better doing so. I didn’t feel as nervous doing things that beforehand I would have been freaking out.*

(E011, Female, 17 years)

### 3.3. Appeal

Participants found several aspects of Entourage appealing. These included the bespoke therapy comics, the accessibility of Entourage and its availability to use as a back-up support when needed, and that it provided relatable and relevant information. The opportunity to learn new skills to manage anxiety, and an openness to try something new to help themselves were themes most often mentioned by participants (*n* = 41).

*I really liked the cartoon comics; they were really good. It’s just like better than just having to read babble, it gives you a realistic live situation and you can really empathise and put yourself in the shoes of the comics, it just gives you a better understanding, seeing an animation play out what I’m thinking. *

(E225, Male, 23 years)

*I think the fact that you can just do it by yourself. Yeah, you don’t have to take yourself anywhere, you can just stay in your pyjamas, you can… just be gentle with yourself. Yeah, to me it was kind of like therapy at home. *

(E031, Female, 17 years)

#### Appeal to Young Males

Participants were specifically asked about what they considered important for encouraging young men to join a platform like Entourage. Overall, some were uncertain what would appeal most to young males (*n* = 18). However, suggestions made only by male participants included: a desire for mental health content delivered by male role models (e.g., musicians; *n* = 2), a familiar platform that was similar to social media or included gamification (*n* = 6), the importance of relatable and authentic content (*n* =5), and evidence-based content (*n* = 6). Two males suggested that having the platform recommended to them by a peer or someone trustworthy would help them to join the platform. In addition, reinforcing the safety of the platform (i.e., a non-judgemental, non-stigmatising space) and normalising the experience of mental health appeared to be important factors, and these were suggested by participants across genders.

*I think stigma is the biggest thing. But implementing it is the hardest thing. The only thing you can do is just talk, just get the conversation going…other than getting … people, not even like athletes or anything, just people that have their lives together, you know what I mean? Talking about it saying, talking about their experiences, I imagine that definitely helps.*

(E018, Male, 24 years)

*Probably just to reinforce the safety of it, you know what I mean? Like, that is a safe place to be and there’s no judgment or prejudice or anything like that. *

(E230, Male, 23 years)

### 3.4. Disengagement

Disengagement related to reasons young people provided for not using Entourage in an ongoing manner. Participants highlighted that they did not use Entourage consistently throughout the 12-weeks. Reasons for this included: non-user friendliness of the platform, feeling like they did not have enough time to fully utilise Entourage, a disinterest in certain elements such as the social network, and anxiety impacting their ability to engage. For example, one participant stated,

*Giving people the opportunity to connect with other young people and that sense of community for working to better themselves; yeah, I’m not sure if it was super helpful for me. I think that’s particularly an age thing. Just because I’m right at that upper end of the age thing. I think it’s probably suited for a little bit younger than I am.*

(E231, Male, 25 years)

Some participants (*n* = 9) had hoped to connect with other young people on Entourage and felt disengaged and disconnected when this did not occur. Important suggestions were offered to overcome the fear of initiating contact with another young person on the site. For example, it was suggested that providing more structure and encouragement to communicate with other users by randomly matching individuals up to talk with each other, scheduled online group discussions via video, or providing opportunities to meet in person would help overcome online social anxiety.

F*irst thing, offering ways to encourage talking to other people. Encouraging more interaction between everyone. E.g. an optional thing where you can join a group to do something online, which could encourage people to talk about that thing outside of the group. Real life events would also help.*

(E214, Male, 19 years)

*When I was talking in one of my headspace sessions the other week about Entourage, we mentioned about how we are all so anxious and afraid about talking online it is difficult to contribute on a site like Entourage, like if there was something to prompt engagement without feeling too exposed. Some anonymous and like an icebreaker sort of forum. Some kind of bridge to connect with other people.*

(E217, Female, 18 years)

### 3.5. System Improvement

System improvement related to young people’s views on ways in which the platform could be enhanced. Many participants found the platform to have some non-user-friendly aspects—such as a “clunky” interface. Participants reflected that they had difficulty navigating to specific content of interest or returning to where they had previously finished. Some found this experience “frustrating” or “confusing”. Some users also wished to be able to view a summary of their completed modules or therapy content and to be able to reflect on their progress. A number of young people (*n =* 12) highlighted that a smartphone application (app) would be an improvement in terms of being easier to access and more likely to encourage regular use (Entourage is available as a web-based app but not a native app). The overall sentiment of feedback from individuals on Entourage was that not all aspects would appeal to everyone, and each individual has different needs.

*Regarding what wasn’t helpful—it wasn’t as though Entourage set me back or had a negative impact, some parts of it just weren’t for me.*

(E013, Male, 16 years)

While not all aspects of Entourage were relevant or appealing to everyone, it was recommended that allowing individuals to choose or tailor their own experience would be an improvement.

*I think keep it all, because not everyone might, specifically, want to do this. Just have it all different ways of addressing the same thing, just all different versions. Just keep it all, I think, can be so helpful. *

(E033, Male, 22 years)

## 4. Discussion

This study used content analysis of qualitative interview data to examine the experiences of 70 young people who used the novel digital intervention for social anxiety, Entourage, with the aim of using insights generated by participants to improve future digital interventions. Results indicated connection with others to be one of the biggest benefits, social anxiety interventions to be both appealing and helpful, suggested reasons for disengagement from the platform and recommendations for future improvement.

### 4.1. Social Anxiety and Engagement

Young people with social anxiety often experience loneliness and social disconnectedness, which has been found to be a maintaining factor of the disorder [23]. One of the most beneficial aspects of Entourage reported by young people was the ability to read others’ experiences of social anxiety and mental ill-health. This provided reassurance that these experiences were common and normal for young people and may have reduced feelings of loneliness. However, many young people reported that while they found this very beneficial, they did not themselves connect or reach out to other members of the platform. Some indicated that this was due to a disinterest in the social networking aspect of Entourage, while others suggested that their social anxiety symptoms stopped them from socially engaging with others on Entourage. It is possible that those who reported a disinterest in the social network of Entourage may have purposefully avoided engaging to remove the possibility of rejection or negative evaluation by their peers; a key aspect of SAD. Previous research indicates that young people with social anxiety can have difficulty fully engaging in traditional face-to-face therapy which is thought to be influenced by symptoms of the disorder itself such as fear of negative evaluation (e.g., by their clinician or therapist) and embarrassment [7]. Therefore, it is likely that the same experience occurs in a digital setting, particularly one that includes a social networking component, and that elements designed to alleviate anxiety (such as anonymity) were not enough to overcome the debilitating nature of social anxiety disorder. Research suggests “lurking” is a widespread phenomenon in online communities anyway, and that up to 90% of online participants do not regularly contribute to discussions, 9% do sometimes and 1% contribute regularly [44]. However, previous findings that have shown young people with social anxiety are more likely to “lurk”, or passively engage on social networking sites, and that fear around public speaking could extend to the “public” nature of newsfeeds on social networks [45]. This may provide further context for disengagement by users if the amount of regular contributions by participants is even lower than 1% in a sample size that is small to begin with. However, features on Entourage that were less “public” than the newsfeed, such as Talk It Out group problem solving and private chat functions with peer and clinical moderators, were positively engaged with by young people on Entourage. A recent qualitative analysis of a MOST platform for young people with first episode psychosis similarly found social anxiety to be a key interfering factor in young people’s use of the platform [46]. Therefore, addressing SAD factors that interfere with engagement with suggestions provided by participants of the current study may help boost engagement and increase benefits for young people involved in digital interventions. To overcome inhibition of initiating social contact with others online, it was suggested that more structured encouragement or support to do so would be beneficial such as being “matched up” with another participant to chat with, moderated online group video calls and even opportunities to meet in real-life. Given that a quarter of participants reported feeling inhibited by anxiety, improving opportunities to overcome this anxiety and connect with others is an important consideration for future digital interventions. Similarly, understanding how young people are implementing skills learned on Entourage via bespoke software features that seek quantitative information from participants along with qualitative data gathered via interviews or focus groups post-intervention. Making it easier to connect with others (e.g., by facilitating interactions) and regular opportunities for doing so (such as scheduled group video calls or in-person meet ups) would require functions to be built into existing platforms that would allow video conferencing. In addition, further considerations for staff monitoring of private chats, privacy and safety would all need to be taken into account. The feasibility and sustainability of organizing ongoing in-person social meet-ups remains unknown and is an area needing further research.

### 4.2. Gendered Experiences

The results indicated minimal gender differences regarding appealing and helpful aspects of the Entourage platform. This also reflects quantitative outcomes of Entourage (published elsewhere) which showed no differences between genders in usage levels or clinical outcomes [30]. Ideas of trustworthiness, peer recommendations, and evidence-based content were suggested by male participants when asked about factors that could facilitate young men’s involvement in digital platforms like Entourage. This may suggest that males required a greater sense of trust or familiarity before engaging, although this was based on a small number of respondents and further exploration of this hypothesis would be beneficial for future research. Our current of understanding of help-seeking tendencies suggests that males are more apprehensive about help-seeking than females, and understanding why is important for both digital and traditional therapies [47]. While it is important to take into account these individual experiences, overall, the results suggest that the program was equally able to cater to the needs of all participants; with approximately similar numbers of males and females participated in the overall pilot study and subsequent qualitative interviews [30]. Previous research has shown that male participants have been more difficult to engage in digital interventions [24], and the current results may be due to the inclusion of young males with lived-experience of social anxiety in the development stage of the project which has likely made the platform more balanced in its appeal to different gender populations. This further supports findings that including consumers in the design of digital interventions is particularly important for acceptability and uptake of such programs [32]. In addition, further research on the experience of gender non-binary and transgender young people would be highly beneficial, as these groups tend to be over-represented in this area [48].

### 4.3. Individual Needs

When providing feedback on their experience of using Entourage, many participants reported that not all aspects of the program were relevant to them. In fact, nearly half of participants suggested that Entourage could be “useful to different people in different ways”. In addition, it was highlighted that some individuals only used Entourage when distressed or feeling down and seeking additional support, but otherwise did not use the platform. The average number of logins by participants was 17.8 times across the duration of the intervention, with an average of 17.2 therapy modules completed and 0.89 social networking posts written [30]. What this means for our understanding of digital intervention usage is that aiming for 100% completion of therapeutic content for best clinical outcomes may not be appropriate or beneficial. Aiming for “effective” engagement rather than simply more engagement may be a more valuable approach; with “effective” defined as sufficient engagement to achieve intended outcomes [49]. This suggests that the “dose” of therapy needed for clinical effectiveness is unlikely to be a one-size-fits-all, and individuals will have differing needs in this regard; aligning with “good enough level” models of change in traditional therapy [50]. Improving the desirability to engage in a platform like Entourage should still be a priority, but this finding highlights the importance of remembering that participants have their own individual needs, preferences and expectations of engagement in digital interventions and the importance of determining the level of support a young person needs at a specific point in time. Importantly, the clinical outcomes of the study (published elsewhere [30]) indicated significant and meaningful improvement on a broad range of symptoms such as social anxiety, loneliness, and social disconnectedness, with no differences identified between genders; this suggests “effective” engagement was achieved. An overarching sentiment raised by participants was that all components of Entourage, such as peer-to-peer networking, therapeutic content and peer and expert moderation, should be continued (with improvements to user-friendliness) to allow individuals to choose what may be helpful to them. This suggests that there is unlikely to be a platform that perfectly suits the needs of all young people accessing it but providing a range of options in terms of information and therapy delivery, opportunities for social connection and practicing learned skills is important to consider.

### 4.4. Study Limitations and Future Directions

While the feedback obtained from a large proportion of study participants was valuable, the interviews were limited in length and at times lacked a depth of exploration of experiences. Possible reasons for this include time-limitations to complete interviews at the conclusion of the follow-up assessment as most appointments were required to be completed within a one-hour session, in addition to social anxiety symptoms limiting participants’ willingness to elaborate (e.g., potentially due to perceived fear of negative evaluation by the researcher). This has likely limited the richness of conclusions drawn in the present study. In addition, to avoid time burden on participants a formal diagnostic assessment of SAD was not used. We recognize this as a significant limitation and may limit the generalizability of the findings. The present qualitative content analysis also reported summary quantitative data (e.g., totals). We acknowledge that this approach has shortcomings, including potential for data misinterpretation. Future research with the Entourage platform should use a range of methodologies to ensure robust and reliable findings. Future studies incorporating qualitative feedback alongside quantitative assessments may seek to complete a smaller subset of interviews with a focus on depth of information rather than breadth. Another limitation evident during analysis was the use of multiple researchers conducting follow-up interviews. The semi-structured nature of the interviews meant that interviewers were free to ask additional questions or explore answers at their discretion. This, however, resulted in some inconsistencies in questioning across interviews. For example, a key question relating to gender and experience: “What do you think is the single most important thing that a project like Entourage could do to get *guys* to join? [or insert other pronoun here for other genders]” was sometimes transposed to “what do you think is the most important thing a project like Entourage could do to get *people* to join?” This omission potentially misses important information about gendered experiences of using the platform and opportunities for future improvement. However, majority of participants were asked about young men specifically and due to the large sample size, an acceptable amount of data was collected on this topic.

It is likely that experiences of the platform differed by age of participants and severity of social anxiety as a result of different needs and expectations at different developmental stages. As the research question of the current study was focused on gendered experiences, age and anxiety severity were not analysed, however these factors should be considered in future evaluations. Entourage provides an accessible, online, evidence-based platform that young people can use as an adjunct to face-to-face therapy or while waiting for sometimes lengthy periods to access community mental health services. Therefore, further information on particular age-groups, settings or symptom severity would be useful for widening the population that may benefit from the targeted support offered by an intervention like Entourage. Future research should seek to incorporate findings from the present study to determine whether this improves appeal and effectiveness of future digital interventions. Important aspects include involving consumers in the design process, actively assisting individuals to overcome social anxiety symptoms to engage with others and allowing individuals to tailor the program to their own needs. Furthermore, an important consideration when determining individual needs would be to collect reliable outcome data throughout the intervention, rather than just at pre- and post-intervention, to identify early response or deterioration during treatment. This will enable moderators to further tailor the support provided to young people on Entourage. Additionally, including mixed methods designs to rigorously evaluate digital interventions should be a common feature of this research. Given that Entourage was piloted within the Australian context in a relatively unique model of care known as headspace developed by McGorry et al. [51], to better understand the generalizability of the findings, piloting the platform in primary care settings internationally that offer different models of care for young people could be a next step. Finally, further exploration should be undertaken related to factors that may impact treatment engagement in SAD, such as embarrassment or shame [52], and the potential role of developing skills in self-compassion to manage these states [53] In particular, there may be a role for future digital interventions to address these domains, with the aim of bolstering functional and symptomatic recovery for young people experiencing SAD.

## 5. Conclusions

This study presents valuable information to guide future digital interventions, with a particular focus on young people with social anxiety. It was evident that reading others’ experiences of anxiety was very important to young people by normalising and reducing the loneliness associated with their own experiences; which are important protective factors. The availability of psychoeducational material on anxiety and practical strategies for managing their symptoms was considered helpful, as well as the option to use different aspects of the platform in a self-directed way. It may be the case that effective treatment outcomes are not dependent on full completion or engagement with the intervention, however, more investigation is needed in this area. No overt gender differences were found in the appeal or experience of using Entourage. This suggests that Entourage was successful in its aim of improving the engagement of young males in treatment, which is an important goal to improve the wellbeing of young people in general, but young men in particular. The current study highlights that digital interventions can provide accessible, appealing and effective treatment for young people with social anxiety and the important role these interventions can play in reaching those who have difficulty accessing traditional services, particularly young men.

## Figures and Tables

**Table 1:** Content Analysis Procedure Adapted from Erlingsson and Brysiewicz [40].

Phase.	Examples of Procedure for Each Step
1. Familiarisation with the data	Transcribe data; read and re-read transcripts; document reflective thoughts; store raw data in well-organised archives
2. Identify and Condense Meaning Units	Divide the text into meaning units; condense meaning units further keeping the central meaning intact; researcher triangulation
3. Formulate Codes	Generate a coding framework from initial meaning units; Code features of the data systematically across the dataset, collate data relevant to each code; researcher triangulation; supervision; document all meetings and decisions
4. Develop Categories	Organise codes into potential categories, gather all data relevant to each potential category; keep detailed notes about development and organisation of concepts
5. Review Categories	Researcher triangulation; Supervision and vetting of categories; check if categories are consistent in relation to coded extracts and entire data-set; team consensus of categories
6. Produce Report	Final opportunity for analysis; select appropriate extracts; thick descriptions of context; description of coding and analysis; produce report that includes theoretical, methodological and analytical choices throughout entire study

**Table 2:** Summary of Participant Experiences of Using the Entourage Platform.

Category	Number of Transcripts Mentioned in (Males Mentioned) *n* = 70			Category	Number of Transcripts Mentioned in (Males Mentioned) *n* = 70		
Connection	*n*	% Total	% Males	Appeal (Young Males)	*n*	% Total	% Males
Shared experiences normalised own experiences	33 (17)	47.1	51.5	Uncertain what appeals to young males	18 (9)	25.7	50.0
Increased confidence to talk or connect with other people	32 (15)	45.7	46.9	Address stigma and normalise help-seeking	15 (9)	21.4	60.0
The environment felt supportive	27 (11)	38.6	40.7	Accessible online	14 (8)	20.0	57.1
Connecting with other users on Entourage	26 (12)	37.1	46.2	A safe and trustworthy environment	14 (7)	20.0	50.0
Therapist support was helpful	21 (12)	30.0	57.1	Familiarity is appealing to young males (e.g., games)	6 (6)	8.6	100.0
The opportunity to connect with others was appealing	21 (10)	30.0	47.6	Evidence-based content	6 (6)	8.6	100.0
Being able to discuss and problem solve issues as a group	20 (7)	28.6	35.0	Authentic, relatable content	5 (5)	7.1	100.0
Entourage helped to share problems and be more open	15 (9)	21.4	60.0	Support from other male peers	5 (2)	7.1	40.0
Entourage has not enhanced social environment	14 (6)	20.0	42.9	Privacy	4 (1)	5.7	25.0
Entourage helped to form new relationships	6 (2)	8.6	33.3	Having real people on Entourage	3 (3)	4.3	100.0
Peer moderator support was helpful	3 (1)	4.3	33.3	Mental health content from role models	2 (2)	2.9	100.0
Having real people as support on Entourage was helpful	1 (1)	1.4	100.0	Recommended by a peer or someone trustworthy	2 (2)	2.9	100.0
**Appeal (Overall)**				**Anxiety Management**			
Open to trying something new to help themselves	41 (20)	58.6	48.8	Anxiety psychoeducation and management strategies were helpful	57 (29)	81.4	50.9
Opportunity to learn anxiety management techniques	41 (17)	58.6	41.5	The therapy modules (steps) were beneficial	49 (25)	70.0	51.0
Easy to use and accessible when needed	29 (11)	41.4	37.9	Available as a back-up support to use when needed	19 (9)	27.1	47.4
Content was relatable and appropriate	25 (12)	35.7	48.0	Entourage helped to reduce anxiety symptoms	17 (8)	24.3	47.1
Worked reliably and as it was expected to	23 (14)	32.9	60.9	The mindfulness tracks were beneficial	11 (5)	15.7	45.5
Entourage was a desirable platform	20 (9)	28.6	45.0	Notifications were helpful as a reminder	10 (6)	14.3	60.0
Entourage appeared like a safe environment	4 (0)	5.7	0.0	Reading other’s experiences of anxiety was reassuring	10 (4)	14.3	40.0
Entourage was recommended to them	18 (3)	25.7	16.7	Entourage could be used for solving problems	9 (3)	12.9	33.3
Could be used in a self-directed way	13 (4)	18.6	30.8	Reinforced strategies learned in face-to-face therapy	6 (2)	8.6	33.3
Interested in helping others through research or on the platform	11 (7)	15.7	63.6	Entourage provided the opportunity to practice skills	6 (2)	8.6	33.3
Anonymity	8 (3)	11.4	37.5				
Having real people on Entourage was appealing	5 (2)	7.1	40.0				
Reimbursement for participating	2 (1)	2.9	50.0				
**Disengagement**				**System Improvement**			
Did not have enough time to fully engage with Entourage	24 (12)	34.3	50.0	Useful to different people in different ways	31 (15)	44.3	48.4
Entourage was not very user-friendly	18 (9)	25.7	50.0	Could be more user-friendly (e.g., fewer system glitches)	25 (15)	35.7	60.0
Anxiety stopped them from engaging	18 (1)	25.7	5.6	Increased study promotion	19 (9)	27.1	47.4
The social element of Entourage was not relevant	15 (8)	21.4	53.3	More encouragement or assistance to connect with other users would have been helpful	15 (6)	21.4	40.0
Not interested in social aspect of Entourage	14 (6)	20.0	42.9	Uncertain what could have improved Entourage	15 (5)	21.4	33.3
The information on Entourage was not relevant	14 (2)	20.0	14.3	Would be more user-friendly as an application (app)	12 (8)	17.1	66.7
Did not feel connected to other users on Entourage	9 (4)	12.9	44.4	Notifications could be improved	8 (5)	11.4	62.5
Too much information on Entourage	8 (5)	11.4	62.5	Tailor the platform for individual preferences	8 (4)	11.4	50.0
Forgot to use Entourage	8 (3)	11.4	37.5	Information on Entourage could be too complicated	4 (2)	5.7	50.0
There was not a lot of activity occurring on Entourage	8 (3)	11.4	37.5	Some content on Entourage was too simple	3 (1)	4.3	33.3
Notifications from Entourage could feel overwhelming	7 (4)	10.0	57.1	More gamification	2 (1)	2.9	50.0
Preferred in-person support	7 (4)	10.0	57.1	More rewards for engaging in content	2 (0)	2.9	0.0
Avoided using Entourage (e.g., when feeling guilty or mental health had worsened)	7 (3)	10.0	42.9				
Did not get support or answers as quickly as desired	3 (1)	4.3	33.3				
Distrusting because had not met anyone in real life	2 (0)	2.9	0.0				

## Data Availability

Due to research ethics approval, data is not publicly available. The corresponding author can be contacted for further information.

## Appendix A

**Table A1:** Entourage Study Inclusion and Exclusion Criteria.

Participant Inclusion Criteria
Aged 12–25 years inclusive; Recent symptoms of social anxiety in the past 4 weeks indicated by a score of at least 30 on the Leibowitz Social Anxiety Scale; Are attending one of four headspace centres across north-western Melbourne; Are able to give informed consent; Have regular and ongoing use to the internet/a smartphone.
Participant Exclusion Criteria
Presence of an intellectual disability; Not able to converse in, or read English; Presence of comorbid physical health conditions requiring a high level of medical care; Current diagnosis of a schizophrenia spectrum or psychotic disorder.

## Appendix B

**Table A2:** Researcher information guided by CORE-Q guidelines [38].

Researcher	BOB	MW	LV
Interviews Conducted	28	35	7
Credentials	BA (Hons)	BA (Hons)	MSOCWK
Gender	Female	Male	Non-Binary
Experience and Training	Training completed on Entourage interview schedule.	Training completed on Entourage interview schedule in addition to previous qualitative interviewing experience.	Training completed on Entourage interview schedule in addition to previous qualitative interviewing experience.

Bridget O’Bree (BOB); Michael Wilson (MW); Lee Valentine (LV); Bachelor of Arts (BA); Master of Social Work (MSOCWK).

## Appendix C

**Table A3:** Semi-Structured Interview Schedule and Guiding Research Questions.

Research Questions	Interview Questions
How did the project’s recruitment strategies influence the uptake and use of the project, and how did different populations of males respond?	Why did you join the Entourage project?
How did the gender sensitization of the project influence the ways that participants engaged with the program, and how did this differ by male characteristics?	What do you think is the single most important thing that a project like Entourage could do to get guys to join? [or insert other pronoun here for other genders] Do you think that Entourage hit the mark as far as [that thing]?
How did aspects of project implementation influence participant engagement with the project?	What kept you coming back to Entourage?
How did males interact with the project in ways that mobilize or constrain social connectedness or other outcomes, and how did this vary by context?	What would you say was the biggest benefit for you from the Entourage project? Can you give me an example of a time when Entourage helped you? What would you say was least helpful in the project? Can you give me an example of a time when the project did not help you? How has participating in Entourage changed how you connect with other fellas? What aspects of Entourage have helped enhance your social involvement?
What aspects of the project and project at large support the sustainability and scalability of the projects?	What parts of this project do you think should be continued so that other youth can benefit from them? If there was one thing the people running the project could improve, what would that be? For our final question, we’d like to know if there is anything else that you would like to share that we haven’t already asked about?

## References

[1] Burstein M., He J.-P., Kattan G., Albano A.M., Avenevoli S., Merikangas K.R. (2011). Social phobia and subtypes in the National Comorbidity Survey–Adolescent Supplement: Prevalence, correlates, and comorbidity. J. Am. Acad. Child. Adolesc. Psychiatry.

[2] Kessler R. (2003). The impairments caused by social phobia in the general population: Implications for intervention. Acta Psychiatr. Scand..

[3] American Psychiatric Association (2013). Diagnostic and Statistical Manual of Mental Disorders (DSM-5).

[4] Stein D.J., Lim C.C., Roest A.M., de Jonge P., Aguilar-Gaxiola S., Al-Hamzawi A., Alonso J., Benjet C., Bromet E.J., Bruffaerts R. (2017). The cross-national epidemiology of social anxiety disorder: Data from the World Mental Health Survey Initiative. BMC Med..

[5] Stein D.J., Scott K.M., de Jonge P., Kessler R.C. (2017). Epidemiology of anxiety disorders: From surveys to nosology and back. Dialogues Clin. Neurosci..

[6] Aderka I.M., Hofmann S.G., Nickerson A., Hermesh H., Gilboa-Schechtman E., Marom S. (2012). Functional impairment in social anxiety disorder. J. Anxiety Disord..

[7] Nordh M., Vigerland S., Öst L.-G., Ljótsson B., Mataix-Cols D., Serlachius E., Högström J. (2017). Therapist-guided internet-delivered cognitive–behavioural therapy supplemented with group exposure sessions for adolescents with social anxiety disorder: A feasibility trial. BMJ Open.

[8] Griffiths K.M., Walker J., Batterham P.J. (2017). Help seeking for social anxiety: A pilot randomised controlled trial. Digit. Health.

[9] Koyuncu A., İnce E., Ertekin E., Tükel R. (2019). Comorbidity in social anxiety disorder: Diagnostic and therapeutic challenges. Drugs Context.

[10] Nicholas J., Oliver K., Lee K., O’Brien M. (2004). Help-seeking behaviour and the Internet: An investigation among Australian adolescents. Aust. E-J. Adv. Ment. Health.

[11] Renton T., Tang H., Ennis N., Cusimano M.D., Bhalerao S., Schweizer T.A., Topolovec-Vranic J. (2014). Web-based intervention programs for depression: A scoping review and evaluation. J. Med. Internet Res..

[12] Rice S.M., Purcell R., McGorry P.D. (2018). Adolescent and young adult male mental health: Transforming system failures into proactive models of engagement. J. Adolesc. Health.

[13] Ridout B., Campbell A. (2018). The use of social networking sites in mental health interventions for young people: Systematic review. J. Med. Internet Res..

[14] Valentine L., McEnery C., D’Alfonso S., Phillips J., Bailey E., Alvarez-Jimenez M. (2019). Harnessing the potential of social media to develop the next generation of digital health treatments in youth mental health. Curr. Treat. Options Psychiatry.

[15] Grist R., Croker A., Denne M., Stallard P. (2019). Technology Delivered Interventions for Depression and Anxiety in Children and Adolescents: A Systematic Review and Meta-analysis. Clin. Child. Fam. Psychol. Rev..

[16] Podina I.R., Mogoase C., David D., Szentagotai A., Dobrean A. (2016). A meta-analysis on the efficacy of technology mediated CBT for anxious children and adolescents. J. Ration. -Emot. Cogn. -Behav. Ther..

[17] Carpenter J.K., Andrews L.A., Witcraft S.M., Powers M.B., Smits J.A., Hofmann S.G. (2018). Cognitive behavioral therapy for anxiety and related disorders: A meta-analysis of randomized placebo-controlled trials. Depress. Anxiety.

[18] Mayo-Wilson E., Dias S., Mavranezouli I., Kew K., Clark D.M., Ades A., Pilling S. (2014). Psychological and pharmacological interventions for social anxiety disorder in adults: A systematic review and network meta-analysis. Lancet Psychiatry.

[19] Spence S.H., Donovan C.L., March S., Gamble A., Anderson R.E., Prosser S., Kenardy J. (2011). A randomized controlled trial of online versus clinic-based CBT for adolescent anxiety. J. Consult. Clin. Psychol..

[20] Eysenbach G. (2005). The law of attrition. J. Med. Internet Res..

[21] Rickwood D., Bradford S. (2012). The role of self-help in the treatment of mild anxiety disorders in young people: An evidence-based review. Psychol. Res. Behav. Manag..

[22] Naslund J.A., Aschbrenner K.A., Marsch L.A., Bartels S.J. (2016). The future of mental health care: Peer-to-peer support and social media. Epidemiol. Psychiatr. Sci..

[23] Lim M.H., Rodebaugh T.L., Zyphur M.J., Gleeson J.F. (2016). Loneliness over time: The crucial role of social anxiety. J. Abnorm. Psychol..

[24] Clarke A.M., Kuosmanen T., Barry M.M. (2015). A systematic review of online youth mental health promotion and prevention interventions. J. Youth Adolesc..

[25] Alvarez-Jimenez M., Bendall S., Lederman R., Wadley G., Chinnery G., Vargas S., Larkin M., Killackey E., McGorry P.D., Gleeson J.F. (2013). On the HORYZON: Moderated online social therapy for long-term recovery in first episode psychosis. Schizophr. Res..

[26] Gleeson J., Lederman R., Herrman H., Koval P., Eleftheriadis D., Bendall S., Cotton S.M., Alvarez-Jimenez M. (2017). Moderated online social therapy for carers of young people recovering from first-episode psychosis: Study protocol for a randomised controlled trial. Trials.

[27] Rice S., Gleeson J., Davey C., Hetrick S., Parker A., Lederman R., Wadley G., Murray G., Herrman H., Chambers R. (2018). Moderated online social therapy for depression relapse prevention in young people: Pilot study of a ‘next generation’online intervention. Early Interv. Psychiatry.

[28] Alvarez-Jimenez M., Rice S., D’Alfonso S., Leicester S., Bendall S., Pryor I., Russon P., McEnery C., Santesteban-Echarri O., Da Costa G. (2020). A Novel multimodal digital service (moderated online social therapy+) for help-seeking young people experiencing mental ill-health: Pilot evaluation within a national youth e-mental health service. J. Med. Internet Res..

[29] Alvarez-Jimenez M., Gleeson J.F., Bendall S., Penn D.L., Yung A.R., Ryan R.M., Eleftheriadis D., D’Alfonso S., Rice S., Miles C. (2018). Enhancing social functioning in young people at Ultra High Risk (UHR) for psychosis: A pilot study of a novel strengths and mindfulness-based online social therapy. Schizophr. Res..

[30] Rice S., O’Bree B., Wilson M., McEnery C., Lim M.H., Hamilton M., Gleeson J., Bendall S., D’Alfonso S., Russon P. (2020). Leveraging the social network for treatment of social anxiety: Pilot study of a youth-specific digital intervention with a focus on engagement of young men. Internet Interv..

[31] Gleeson J.F., Alvarez-Jimenez M., Lederman R. (2012). Moderated online social therapy for recovery from early psychosis. Psychiatr. Serv..

[32] Stallard P., Richardson T., Velleman S., Attwood M. (2011). Computerized CBT (Think, Feel, Do) for depression and anxiety in children and adolescents: Outcomes and feedback from a pilot randomized controlled trial. Behav. Cogn. Psychother..

[33] Glasgow R.E. (2009). Enhancing the scientific foundation of internet intervention research. Ann. Behav. Med..

[34] Proudfoot J., Klein B., Barak A., Carlbring P., Cuijpers P., Lange A., Ritterband L., Andersson G. (2011). Establishing guidelines for executing and reporting internet intervention research. Cogn. Behav. Ther..

[35] Beattie A., Shaw A., Kaur S., Kessler D. (2009). Primary-care patients’ expectations and experiences of online cognitive behavioural therapy for depression: A qualitative study. Health Expect..

[36] Barker C., Pistrang N., Elliott R. (2015). Research Methods in Clinical Psychology: An. Introduction for Students and Practitioners.

[37] Rice S., O’Bree B., Wilson M., McEnery C., Lim M.H., Hamilton M., Gleeson J., Bendall S., D’Alfonso S., Russon P. (2020). Development of a graphic medicine-enabled social media-based intervention for youth social anxiety. Clin. Psychol..

[38] Tong A., Sainsbury P., Craig J. (2007). Consolidated criteria for reporting qualitative research (COREQ): A 32-item checklist for interviews and focus groups. Int. J. Qual. Health Care.

[39] McEnery C., Lim M.H., Knowles A., Rice S., Gleeson J., Howell S., Russon P., Miles C., D’Alfonso S., Alvarez-Jimenez M. (2019). Social anxiety in young people with first-episode psychosis: Pilot study of the EMBRACE moderated online social intervention. Early Interv. Psychiatry.

[40] Erlingsson C., Brysiewicz P. (2017). A hands-on guide to doing content analysis. Afr. J. Emerg. Med..

[41] Krippendorff K. (2018). Content Analysis: An Introduction to its Methodology.

[42] Liebowitz M.R. (1987). Social Phobia. Mod. Probl. Pharm..

[43] Kroenke K., Spitzer R.L., Williams J.B. (2001). The PHQ-9: Validity of a brief depression severity measure. J. Gen. Intern. Med..

[44] Sun N., Rau P.P.-L., Ma L. (2014). Understanding lurkers in online communities: A literature review. Comput. Hum. Behav..

[45] Bonetti L., Campbell M.A., Gilmore L. (2010). The relationship of loneliness and social anxiety with children’s and adolescents’ online communication. Cyberpsychologybehav. Soc. Netw..

[46] Valentine L., McEnery C., O’Sullivan S., Gleeson J., Bendall S., Alvarez-Jimenez M. (2020). Young People’s Experience of a Long-Term Social Media–Based Intervention for First-Episode Psychosis: Qualitative Analysis. J. Med. Internet Res..

[47] Addis M.E., Mahalik J.R. (2003). Men, masculinity, and the contexts of help seeking. Am. Psychol..

[48] Millet N., Longworth J., Arcelus J. (2017). Prevalence of anxiety symptoms and disorders in the transgender population: A systematic review of the literature. Int. J. Transgend..

[49] Yardley L., Spring B.J., Riper H., Morrison L.G., Crane D.H., Curtis K., Merchant G.C., Naughton F., Blandford A. (2016). Understanding and promoting effective engagement with digital behavior change interventions. Am. J. Prev. Med..

[50] Baldwin S.A., Berkeljon A., Atkins D.C., Olsen J.A., Nielsen S.L. (2009). Rates of change in naturalistic psychotherapy: Contrasting dose–effect and good-enough level models of change. J. Consult. Clin. Psychol..

[51] McGorry P., Trethowan J., Rickwood D. (2019). Creating headspace for integrated youth mental health care. World Psychiatry.

[52] Kealy D., Rice S.M., Ogrodniczuk J.S., Spidel A. (2018). Childhood trauma and somatic symptoms among psychiatric outpatients: Investigating the role of shame and guilt. Psychiatry Res..

[53] Callow T.J., Moffitt R.L., Neumann D.L. (2021). External shame and its association with depression and anxiety: The moderating role of self-compassion. Aust. Psychol..

